# Urinary incontinence and its relation to delivery circumstances: A population-based study from rural Kilimanjaro, Tanzania

**DOI:** 10.1371/journal.pone.0208733

**Published:** 2019-01-23

**Authors:** Gileard G. Masenga, Benjamin C. Shayo, Sia Msuya, Vibeke Rasch

**Affiliations:** 1 Kilimanjaro Christian Medical University College, Moshi, Klimanjaro, Tanzania; 2 Department of Obstetrics and Gynecology, Kilimanjaro Christian Medical Centre, Moshi, Kilimanjaro, Tanzania; 3 Department of Obstetrics and Gynecology, Odense University Hospital, Odense, Denmark; 4 Department of Clinical Research, University of Southern Denmark, Odense, Denmark; University Medical Center Utrecht, NETHERLANDS

## Abstract

**Objectives:**

To investigate the prevalence and risk factors of urinary incontinence (UI), the different UI subtypes and the association between UI and delivery circumstances.

**Design:**

Cross-sectional population-based study conducted in Kilimanjaro Region, Tanzania.

**Participants and settings:**

1048 women aged 18–90 women living in rural Kilimanjaro. Simple random sampling was done to select villages, households and participants. Community health workers helped in identifying eligible women and trained nurses/midwives conducted face-to-face interviews. Data were analysed using descriptive statistics and Univariate and Multivariate logistic regression modelling.

**Results:**

The overall prevalence rate of UI was 42%. When focusing on the different types of UI, 17% of the women had stress UI, 9% had urge UI and 16% had mixed UI. Only one woman (0.1%) with vesico-vaginal fistula was identified. UI was found to be significantly associated with increasing parity (OR = 2.41 (1.55–3.74). In addition, women who in relation to their first delivery had delivered at home or had been in labour for more than 24 hours, had increased adjusted ORs of 1.70(1.08–2.68) and 2.10(1.08–4.10), respectively, for having UI.

**Conclusion:**

UI is common in rural Tanzania and of the subtypes of UI, Stress Urinary Incontinence (SUI) is the commonest followed by Mixed Urinary Incontinence (MUI). Home delivery, prolonged labour and increasing parity especially having 5 or more deliveries are associated with increased risk for developing UI.

## Introduction

Urinary incontinence, defined as involuntary loss of urine, is a common and devastating condition among women [1]. The prevalence of incontinence in women is estimated to be 29%; however the prevalence estimates vary widely from 5% to 71% [2]. This wide range reflects that studies have been performed in different populations where different methodologies have been applied.

There are three major types of incontinence: (i) stress urinary incontinence (SUI), that is involuntary loss of urine during coughing, sneezing, or exerting effort (ii) urge urinary incontinence (UUI) meaning an involuntary loss of urine with sudden desire to void and (iii) mixed urinary incontinence (MUI) which is the combination of SUI and MUI [1, 3, 4]. SUI has been reported to be the most predominant type of incontinence in studies from high-income countries [5]. It is also the most predominant type of incontinence in younger women, whereas UUI and MUI are more prevalent in older women [6–8].

Risk factors for incontinence have been established in studies from high-income countries and they include, pregnancy, labour, vaginal delivery, body mass index, and genetic factors [4, 9]. Other factors are heavy physical functions/activity, diabetes mellitus, hysterectomy, smoking, caffeine intake, urinary tract infection, and exercise [4, 7, 10]. When focusing on low-income countries, a number of Nigerian studies have documented that increasing age and parity are associated with increased risk of incontinence [11–13]. Incontinence is associated with social and economic burden for the women affected and their families [4]. The social consequence of incontinence includes physical and emotional isolation that has a negative impact on quality of life [14].

When focusing on sub-Saharan Africa, a substantial number of studies have been performed to describe and address the problem of vesico-vaginal fistula and the associated urine leakage. In contrast, little attention has been given to address the problem of incontinence. It may be argued that women suffering from vesico-vaginal fistula only represent the tip of the iceberg. Hence many more women exposed to long lasting labour without skilled attendance are at risk of developing incontinence. So far little is known about the prevalence of different types of incontinence and their association with delivery circumstances in a sub-Saharan African context.

Acknowledging the knowledge gap on urinary incontinence in low-income countries, this study aims to examine the prevalence and risk factors of the different types of urinary incontinence among Tanzanian women and describe how delivery circumstances are associated with incontinence.

## Material and methods

### Study setting

This study was part of the PEDITA (Pelvic floor disorders in Tanzania) project which was performed to determine the magnitude of pelvic organ prolapse (POP) and UI among rural Tanzanian women and assess the impact of vaginal pessary treatment. The study was performed in Kilimanjaro region in northern Tanzania.

### Sampling and study population

Multi-stage, random sampling was employed to obtain representative districts, wards, villages, sub-villages and, subsequently, households. As a result, 3 districts out of 7 in Kilimanjaro region were selected randomly. These were Hai, Rombo, and Same districts. For each district, 4 wards were selected, then 5 villages per ward, and 4 sub-villages per village. Finally, systematic sampling was used to select 20 households per sub-village from the village household registers. The female household leader/head who was above 18 years old and not pregnant during the study period was considered eligible for the study. In the case of a household not having an eligible candidate, the next household was opted for.

### Data collection procedures

After one week of theoretical and practical training, retired nurses with field research experience visited the selected households and conducted face-to-face interviews with the female household leaders/heads obtaining data on their socio-demographic and reproductive characteristics. The included women were invited to attend a selected, nearby health centre the following day for a more detailed interview on pelvic floor disorders. At the clinic, a nurse administered a Swahili translated version of the urinary distress inventory (UDI-6) which probes the symptoms of urinary incontinence followed by a pelvic examination. The UDI-6 has proven to be reliable, valid, and responsive [15]. We adopted the definition of incontinence as any complaint of urinary leakage as defined by the International Continence Society (ICS), SUI as urine leakage associated with physical exertion, sneezing, or coughing; UUI as urine leakage associated with urgency, and MUI as having both SUI and UUI complaints [16]. In addition, the nurse measured the women’s heights and weights and obtained their body mass indices.

### Data analysis

Data were entered and analysed using SPSS version 24.0 Inc, Chicago, IL. Simple frequencies were run to describe the socio-demographic and reproductive characteristics of the participants. The chi-square test was used to compare the prevalence of overall, SUI, UUI, and MUI by age groups. To determine risk factors associated with incontinence, we performed both univariate and multivariate logistic regression with 95% confidence intervals. To determine how delivery circumstances (place of delivery and duration of delivery) were associated with the occurrence of incontinence, multivariate logistic regression was applied. Two logistic regressions were performed based on apriori assumptions. In model 1, the association between delivery circumstances and any urinary incontinence, SUI, UUI, and MUI was adjusted for age, parity, BMI, and lifting of heavy objects and in model 2 the association was adjusted for all significant variables in crude analysis, such as age, parity, education level, and place of delivery/ duration of labour.

### Ethical consideration

Ethical approval was obtained from the National Institute of Medical Research and the Ethical Committee at Kilimanjaro Christian Medical University College with certification number 811 of February 11, 2015. All participants were informed in detail about the project and signed an informed consent form before taking part in the study.

## Results

A total of 1195 women were interviewed at home and invited for further interview and pelvic examination the next day. In all, 1063 women came to the clinic and 1048 (87.7%) accepted being re-interviewed and having a pelvic examination performed.

**Fig 1:**
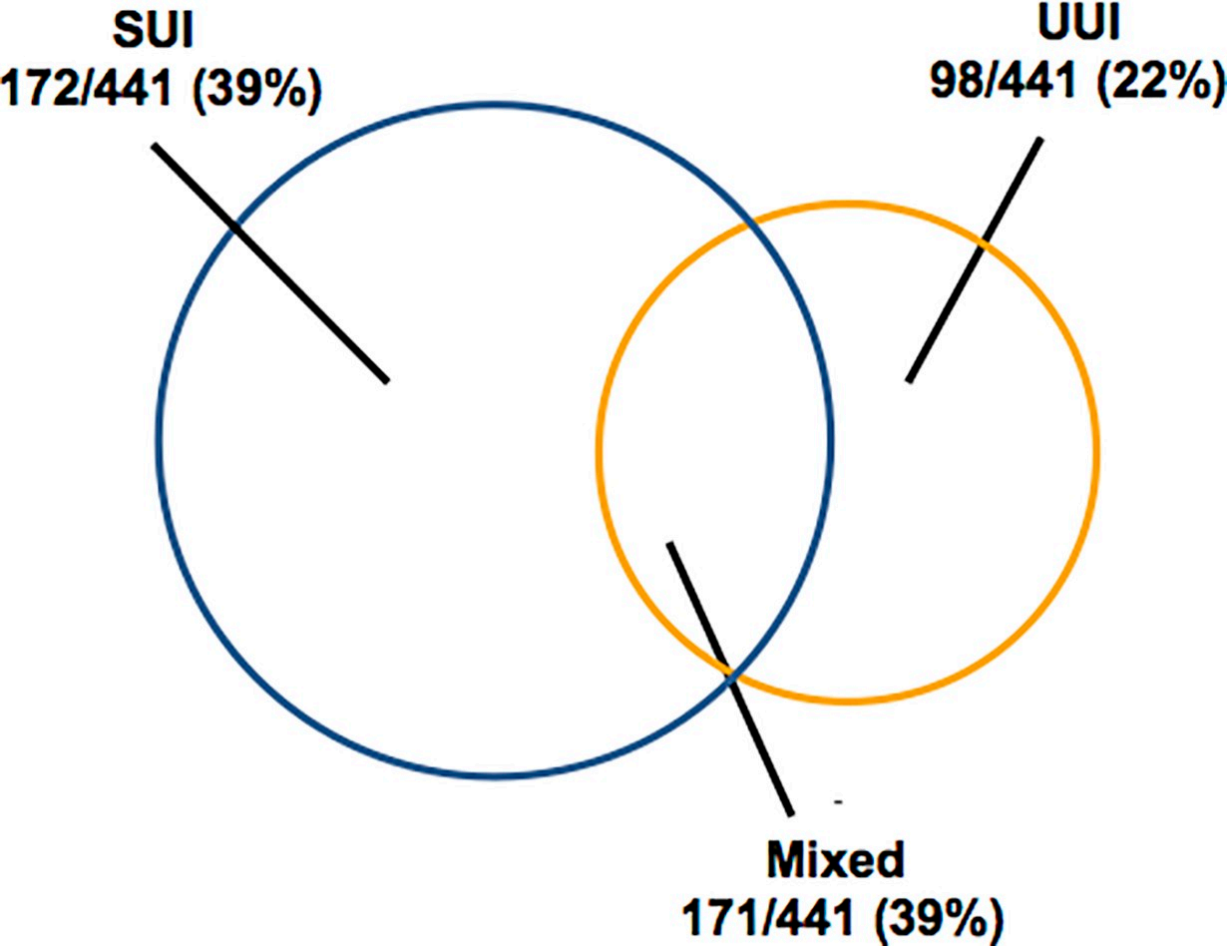
Proportions of stress urinary incontinence (SUI), urge urinary incontinence (UUI), and mixed urinary incontinence (MUI) among 441/1048 women reporting any urinary incontinence.

Some 441/1048 (42.1%) women reported any UI. Among women who stated they had any UI, SUI comprised 39%, UUI 22%, and MUI 39%. Only 1/1048 (0.1%) women complained about constant leakage and were subsequently identified as having a vesico-vaginal fistula (Fig 1).

**Fig 2:**
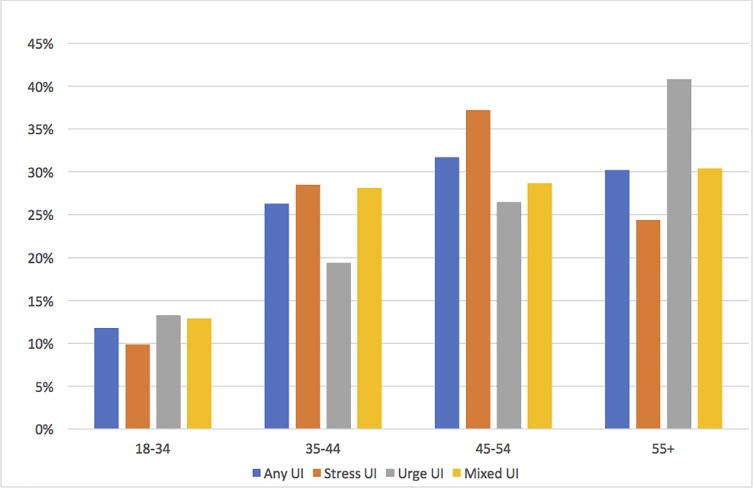
Age and prevalence of subtypes of urinary incontinence.

Overall, the prevalence of UI increased by advancing age, with 48.5% of women aged 55 years and above reporting some form of incontinence. SUI was found to be more common at younger ages with a plateau at the age of 45–50 years, whilst UUI was more common in post-menopausal women (Fig 2). Furthermore, 48.9% of women who had delivered 5 or more times, reported some form of urinary incontinence and 60% of women who had experienced labour for more than 24 hours in their first delivery also reported some form of urinary incontinence (Table 1).

**Table 1:** Background characteristics of the study population and type of urinary incontinence.

Type of UI*	All n = 1048	UI	SUI	UUI	MUI
		n = 441 (%)	n = 172 (%)	n = 98 (%)	n = 171 (%)
Age (n = 1048)					
18–34	165	31.5	10.3	7.9	13.3
35–44	289	40.1	17	6.6	16.6
45–54	320	43.8	20	8.1	15.3
55–90	274	48.5	15.3	14.6	19
Education (n = 1048)					
No formal schooling	219	47.5	13.5	15.1	19.2
Primary	707	42	17.8	7.5	16.5
Secondary and above	122	32.8	13.1	9.8	9.8
Occupation					
Farmer	764	41.5	16.6	8.8	16.1
Business	262	44.3	15.6	11.5	17.2
Others	22	36.4	18.2	4.5	13.6
Heavy work/ day (n = 1048)					
0-1hr	408	38.7	12.7	9.6	16.2
2-4hrs	579	43.9	19.2	9.3	15.5
5+hrs	61	47.7	14.8	8.2	24.6
BMI (n = 1046)					
<24	397	43.1	15.6	10.3	17.1
24–29	356	41	15.7	9.8	15.4
30+	293	42	18.4	7.5	16
Parity (n = 1048)					
0–2	193	25.9	9.3	6.7	9.8
3–4	319	40.4	18.2	6.8	16
5–14	536	48.9	17.9	12.1	18.8
Place 1st del (n = 1047)					
Home	171	40.9	16.4	9.9	14.6
Health center	168	54.8	17.9	11.3	25.6
Hospital	708	39.4	16.1	8.8	14.5
Duration of 1st delivery (n = 1025)					
≤ 24Hrs	985	41.8	16.6	9.4	15.7
> 24Hrs	40	60	15	10	35

*Any urinary incontinence (UI), stress urinary incontinence (SUI), urge urinary incontinence (UUI), and mixed urinary incontinence (MUI)

In the unadjusted analysis of risk factors associated with any UI, SUI, UUI, and MUI, it was found that advancing age and having delivered 3 times or more were associated with increased odds of having any UI as well as increased odds of having any of the subtypes of UI (SUI, UUI, MUI). Furthermore, women who had delivered at home and women who had been in labour for more than 24 hours were more likely to have any UI and as well as the different subtypes of UI (SUI, UUI, MUI). Finally, women who had no formal education were more likely to develop any UI including the different subtypes of UI as compared to those who had secondary education and above (Table 2).

**Table 2:** Univariate analysis showing risk factors associated with any UI, SUI, UUI, and MUI.

	Any UI (n-441)	SUI (n-172)	UUI (n-98)	MUI (n-171)
	COR (95% CI)	COR (95% CI)	COR (95% CI)	COR (95% CI)
Age (years)				
18–34	1	1	1	1
35–44	1.46(0.97–2.18)	**1.16(1.04–2.52)**	1.26(0.79–2.03)	1.42(0.82–2.48)
45–54	**1.69(1.14–2.51)**	**1.8(1.19–2.8)**	1.34(0.8–2.1)	1.39(0.80–2.44)
55–90	**2.05(1.37–3.07)**	**1.8(1.2–2.9)**	**2.1(1.3–3.34)**	**1.89(1.08–3.31)**
Parity				
0–2	**1**	1	1	**1**
3–4	**1.95(1.32–2.89)**	**2.2(1.4–3.4)**	**1.67(1.04–2.6)**	**2.02(1.4–3.57)**
5–14	**2.74(1.9–3.94)**	**2.7(1.8–4.1)**	**2.72(1.7–4.1)**	**2.77(1.63–4.71)**
Place Delivery				
			
Home	1.06(0.76–1.49)	**1.83(1.15–2.9)**	**1.96(1,20–3.20)**	**2.28(1.2–4.08)**
Disp/HC	**1.86(1.32–2.61)**	0.9(0.6–1.3)	0.9(0.62–1.39)	0.97(0.5–1.5)
Hospital	1	1	1	1
Labour				
≤ 24hours	1	1	1	1
> 24hours	**2.08(1.09–3.97)**	**2.25(1.15–4.408)**	**2.58(1.2–5.16)**	**3.24(1.54–6.77)**
BMI (kg/m^2^)				
< 24	1	1	1	1
24–29	0.92(0.69–1.23)	0.92(0.6–1.27)	0.8(0.6–1.2)	0.87(0.58–1.30)
30+	0.96(0.71–1.3)	1.04(0.75–1.44)	0.83(0.58–1.19)	0.91(0.60–1.40)
Hours spent Heavy lifting				
0-1hr	1	1	1	1
2-4hrs	1.24(0.96–1.60)	1.3(0.98–1.72)	1.04(0.7–1.41)	1.04(0.7–1.5)
5+hrs	1.43(0.84–2.46)	1.58(0.89–2.81)	1.47(0.8–2.6)	1.77(0.9–3.47)
Education				
No formal	**1.85(1.17–2.94)**	**1.78(1.05–3.0)**	**2.25(1.3–3.8)**	**2.49(1.23–5.03)**
Primary	1.49(0.99–2.23)	**1.74(1.10–2.75)**	1.4(0.86–2.30)	**1.90(1.02–3.69)**
Secondary+	1	1	1	1
Occupation				
Farmers	1.24(0.52–2.99)	1.14(0.44–2.79)	1.5(0.4–4.6)	1.28(0.36–4.5)
Business	1.39(0.56–3.43)	1.17(0.45–3.30)	1.7(0.56–5.5)	1.43(0.39–5.2)
Others	1	1	1	1
Age 1^st^ Delivery (years)	0.76(0.56–1.04)	1.33(0.9–1.8)	1.32(0.9–1.9)	1.42(0.9–2.1)
< 19				
20–21	1	1	1	1
22+	0.76(0.56–1.05)	1.01(0.7–1.40)	1.00(0.7–1.4)	1.11(0.72–1.70)

However, multivariate analysis revealed that parity, place of delivery and duration of delivery remained significantly associated with any UI and MUI, while only parity was significantly associated with SUI (Table 3).

**Table 3:** Multivariate analysis of risk factors associated with any UI, SUI, UUI and MUI.

	Any UI (n-441)	SUI (n-172)	UUI (n-98)	MUI (n-171)
	aOR (95% CI)	aOR (95% CI)	aOR (95% CI)	aOR (95% CI)
Age				
18–34	1	1	1	1
35–44	0.99(0.63–1.54)	1.34(0.72–2.49)	0.67(0.30–1.50)	0.92(0.51–1.67)
45–54	1.02(0.65–1.61)	1.58(0.85–2.96)	0.69(0.31–1.54)	0.80(0.43–1.47)
55–90	1.16(0.70–1.94)	1.29(0.64–2.62)	1.02(0.44–2.38)	0.97(0.50–1.91)
Parity				
0–2	**1**	1	1	1
3–4	**1.77(1.16–2.70)**	**1.94(1.06–3.55)**	1.02(0.47–2.21)	1.54(0.85–2.80)
5–14	**2.41(1.55–3.74)**	**1.93(1.03–3.59)**	1.81(0.83–3.94)	**1.91(1.03–3.54)**
Place Delivery				
Home	**1.70(1.08–2.68)**	1.00(0.55–1.80)	1.30(0.63–2.66)	**1.95(1.10–3.47)**
Disp/HC	0.95 (0.67–1.34)	0.89(0.55–1.44)	1.02(0.56–1.87)	1.03(0.62–1.70)
Hospital	1	1	1	1
Labour				
≤ 24hours	1	1	1	1
> 24hours	**2.10(1.08–4.10)**	0.92(0.38–2.27)	1.01(0.34–2.96)	**2.71(1.36–5.43)**
Education				
No formal	1.11(0.66–1.87)	0.82(0.40–1.66)	1.04(0.47–2.20)	1.48(0.70–3.12)
Primary	1.18(0.77–1.82)	1.14(0.64–2.03)	0.68(0.34–1.35)	1.57(0.82–3.00)
Secondary+	1	1	1	1

Additional multivariate analyses, were performed to examine the association between delivery factors and different types of UI. A priori adjustment was performed to control for the effect of age, parity, BMI and hours spent in heavy lifting (model 1 in Table 4). In this analysis, delivery factors remained significantly associated with urinary incontinence. Home delivery was found to be a risk factor for development of UI (OR 1.68:1.01–2.66) as well as the different subtypes of UI, SUI (OR1.7:1.05–2.8), UUI (OR 2.0:1.2–3.4), and MUI (OR 2.33:1.26–4.31). Similarly, prolonged labour of more than 24 hours was found to be a risk factor of UI (OR 2.11: 1.08–4.12) and also the subtypes of UI, such as SUI (OR 2.26:1.18–4.5), UUI (OR 2.7:1.3–5.5), and MUI (OR 3.4:1.56–7.40).

**Table 4:** Delivery factors associated with different urinary incontinences.

	Any UI		SUI		UUI		MUI	
Delivery factor	Model 1*	Model 2**	Model 1	Model 2	Model 1	Model 2	Model 1	Model 2
Place of delivery								
Home	**1.68(1.01–2.66)**	**1.73(1.11–2.70)**	**1.7(1.05–2.8)**	**1.78(1.10–2.87)**	**2.0(1.2–3.4)**	**2.06(1.23–3.43)**	**2.33(1.26–4.31)**	**2.35(1.30–4.26)**
Health center	0.94(0.6–1.36)	0.94(0.66–1.34)	0.9(0.6–1.3)	0.93(0.63–1.36)	1.01(0.6–1.5)	0.99(0.65–1.51)	1.00(0.59–1.70)	0.99(0.59–1.64)
Hospital	1	1	1	1	1	1	1	1
Duration of labor								
≤ 24 hours	1	1	1	1	1	1	1	1
≥ 24 hours	**2.11(1.08–4.12)**	**2.21(1.15–4.27)**	**2.26(1.18–4.5)**	**2.30(1.16–4.56)**	**2.7(1.3–5.5)**	**2.84(1.40–5.76)**	**3.40(1.56–7.40)**	**3.37(1.58–7.17)**

* Adjusted for apriori potential confounders of age, parity, BMI, heavy lifting hours

** Adjusted for age, parity, educational level, place of delivery/duration of delivery

Further adjusted analysis, where all the significant variables in the crude analysis were adjusted for, revealed an even stronger association between delivery factors and UI and different subtypes of UI (model 2, Table 4). Women who had their first delivery at home had 1.73 times increased odds of developing IU, 1.78 times increased odds SUI, 2.06 times increased odds of UUI, and 2.35 times increased odds of developing MUI. In addition, women who had experienced a delivery lasting more than 24 hours had 2.21 times odds for developing UI, 2.30 times odds for developing SUI, 2.84 times odds for developing UUI, and 3.37 times odds for developing MUI (Table 4).

## Discussion

In this community-based study conducted among 1048 women, the prevalence of any urinary incontinence was 42%. When focusing on the different types of incontinence, 17% of the women had SUI, 9% had UUI, and 16% had MUI. We identified only one woman with proven vesico-vaginal fistula, giving a fistula prevalence rate of 0.1%. Urinary Incontinence was found to be significantly associated with increasing parity, home delivery, and prolonged labour.

One of the main strengths of this study is that it is based on a large sample size where women were selected through multi-stage random sampling. In addition, 1048 of the 1195 invited women accepted participation. The results may therefore be considered representative for the general population of women in rural Kilimanjaro. Acknowledging that urinary incontinence is a complex topic to study, we used a Kiswahili translation of the widely used UDI-6 questionnaire when aiming at assessing prevalence of the different types of urinary incontinence [17]. The translation process was performed according to a standardized guideline [18]. Since incontinence is considered a highly stigmatizing condition in Tanzania, where neither women nor healthcare providers talk about the condition, there may be a risk of information bias. To ensure optimum accuracy of information on urinary incontinence symptoms, we used thoroughly trained elderly nurses as research assistants, who were used to obtaining information on sensitive topics. In addition, we conducted a pilot study on 20 randomly selected women using the Kiswahili version of the UDI-6 that further enforced the confidence of the nurses in extracting UI-specific, sensitive information from the women. However, we were not able to validate the UDI-6 in our setting before collecting data due to constraints in resources, which is a limitation of our study.

In the current literature, the overall prevalence of incontinence varies from 5% to 71% [2]. We found the prevalence of UI to be considerably higher than prevalence rates reported in studies from other low-income countries such as Ethiopia (7%), Nigeria (2.8–12.2%), and Pakistan (11.5%) [11, 13, 19, 20]. Our findings, are however comparable with studies from Denmark and Germany that have reported prevalence rates of 46.4–48.3% [5]. The most common incontinence subtype in our study was SUI, which is in agreement with findings from both high-income countries and low-income countries [5, 19, 21]. Due to the intimacy of the topic, there is a greater risk of underreporting of incontinence symptoms. We believe that the high prevalence rate of incontinence found in our study reflects the study condition: the interviews were conducted in private surroundings by a team of well-trained, experienced empathetic female nurses who are used to talk openly about sensitive issues, which created an atmosphere of confidentiality.

We found the prevalence of incontinence generally increased as age increases until age 55 years or more where a slight drop in prevalence rate was observed. SUI prevalence peaked at age 45–54 years and then dropped with further increase in age. In contrast, UUI increased as age increased and was the most prevalent type of incontinence among women aged 55 years and above. The observed decrease in SUI after menopause may reflect declining physical activity and an associated decrease in SUI episodes. Additionally, it may also be an expression of the dynamics between the different types of incontinence whereby women with SUI may develop UUI as they age. This can explain the postmenopausal decrease in SUI and the observed subsequent increase in UUI and MUI, an assumption that is supported by an Australian cohort study observing that incontinence is a highly dynamic clinical condition [22].

A strong association between increasing parity and incontinence was also found. Women who had delivered 3–4 times had 1.95 times increased odds for developing any type of incontinence and women who had delivered 5+ times had 2.74 times increased odds for developing any UI. Other studies have similarly found that increasing parity is associated with an increasing risk of subsequent incontinence [23, 24]. When looking at the different types of incontinence, similar associations between increasing parity and SUI, UUI, and MUI were found. The association between increasing parity and UUI is in contrast to other studies that have documented that parity is only associated with SUI or MUI but not with UUI [23, 25]. Our findings may reflect the previously mentioned dynamics among the incontinence subtypes, where women of high parity, who were earlier bothered by SUI only, later on develop UUI and MUI, which may mask their initial SUI. Alternatively, it may reflect that, despite thorough training of our research assistants, we did not manage to teach them how to distinguish between the different types of incontinence and how to explain it to the women they interviewed. Evaluating the responsiveness of the Kiswahili version of the UDI-6 would further increase the strength of our findings.

We also found a strong association between place and duration of first delivery, where women who had delivered at home and women who had been in labour for more than 24 hours had increased ORs of 1.7 and 2.2, respectively, for having urinary incontinence. In low-income countries, it is well known that women who are delivered by unskilled attendants are at an increased risk of obstructed labour and vesico-vaginal fistulas [26–28]. In contrast, little is known about the association between unskilled delivery attendance, prolonged labour, and risk of urinary incontinence in low-income settings. However, studies from high-income countries have documented that women experiencing prolonged labour have an almost two times/almost double increased risk of urinary incontinence [2, 29, 30]. The hypothesis behind the association between prolonged labour and incontinence is that the second stage of labour may lead to permanent nerve damage and weakening of the pelvic floor muscles and fascial support (endopelvic, urethra-pelvic and vesico pelvic) of the urethra. This can eventually lead to urinary incontinence.

We identified only one woman with vesico-vaginal fistula, equivalent to a prevalence rate of 0.1%. We found a low prevalence rate of vesico- vagina fistula in contrast with other studies that have reported prevalence rates of 0.4–8% [31, 32]. The difference in prevalence rates could be explained by the fact that the present study was conducted in Kilimanjaro region where health facility deliveries are common as 90% of women report delivery with a skilled birth attendant [33]. Therefore, the women in our study might not have been exposed to obstructed delivery to the same extent as women who are living in more rural areas. In addition, during the past years, more resources were allocated to programmes addressing the problem of vesico-vaginal fistulas in Tanzania. Surgeons have been trained in fistula repair surgery in Kilimanjaro region as well as in other regions of Tanzania and repair of vesico-vaginal fistulas are offered for free. These initiatives may also have resulted in a decreased number of fistula cases in our study population.

In Tanzania, as well as in other low-income countries where a significant proportion of women still give birth at home, preventive strategies to control incontinence should be considered. The best preventive strategy is to ensure that women have access to skilled delivery attendance. In this connection, it is important that the delivery attendants are trained in best practices in relation to the second stage of labour. Best practice includes allowing the uterus contractions to push the foetus down the birth canal and providing perineal support while the foetal head is being delivered [34, 35]. If applied properly, these procedures may help prevent tearing and damage of the pelvic floor. In addition, focus should be placed on the beneficial effect of pelvic floor training during pregnancy and post-partum.

In conclusion, this study has demonstrated that urinary incontinence is common in rural Tanzania and associated with increasing parity. In addition, home deliveries and prolonged labour are risk factors of urinary incontinence. To address the problem of incontinence, it is important that attention is given to increase knowledge and awareness of the condition. Due to high cost of medicines and limited availability of surgeons, reasonable access to medical treatment and surgical repair services for urinary incontinence is not an option for the majority of Tanzanian women. It is therefore of paramount importance that health workers are trained in available low-cost conservative approaches to treat and prevent urinary incontinence; these involve behavioural changes, pelvic floor exercises, and fitting of urinary incontinence pessaries.

## Supporting information

S1 Dataset UI prevalence and delivery circumstances in Kilimanjaro_PLOS ONE. (SAV)

S1 Questionnaire English version of urinary distress inventory -6(UDI-6). (DOCX)

S2 Questionnaire Kiswahili version of the urinary distress inventory-6 (UDI-6). (DOCX)
