# Supplementary material for: Urinary incontinence and its relation to delivery circumstances: A population-based study from rural Kilimanjaro, Tanzania
Source: PLoS One. 2019 Jan 23;14(1):e0208733. doi: 10.1371/journal.pone.0208733 (PMC6343883; doi:10.1371/journal.pone.0208733)
Supplement: S1 Questionnaire — (DOCX) [file pone.0208733.s002.docx]

**Pelvic floor disorders among women in Kilimanjaro Region, Tanzania**

*PEDITA*

| Date [ ][ ] [ ][ ] [ ][ ] (ddmmyy)) Health facility …………………………………  Registration no……………..………….(from appointment card) Interviewer…………………………………….. |
| --- |

**PART I Symptoms of urinary incontinence (UDI6)**

|  | No, not at all | Somewhat | Moderately | Yes, very much |
| --- | --- | --- | --- | --- |
| 1. Are you bothered by frequent urination? |  |  |  |  |
| 2. Are you bothered by involuntarily loss of urine? |  |  |  |  |
| 3. Do you experience urine leakage related to the feeling of urgency – a strong sensation of needing to go to the bathroom? |  |  |  |  |
| 4. Do you experience urine leakage related to coughing, sneezing and physical activity? |  |  |  |  |
| 5. Do you have any trouble emptying your bladder? |  |  |  |  |
| 6. Do you have any pain while urinating? |  |  |  |  |
