# Supplementary material for: Urinary incontinence and its relation to delivery circumstances: A population-based study from rural Kilimanjaro, Tanzania
Source: PLoS One. 2019 Jan 23;14(1):e0208733. doi: 10.1371/journal.pone.0208733 (PMC6343883; doi:10.1371/journal.pone.0208733)
Supplement: S2 Questionnaire — (DOCX) [file pone.0208733.s003.docx]

**Dalili za kutokwa na mkojo bila ridhaa (UDI-6)**

|  | Hapana, sivyo kabisa | Pengine | Kwa wastani | Ndio, sana kabisa |
| --- | --- | --- | --- | --- |
| 1. Je, unasumbuliwa na kupata haja ndogo mara kwa mara? |  |  |  |  |
| 2. Je, unasumbuliwa na haja ndogo kutoka bila idhini yako? |  |  |  |  |
| 3. Je, unatokwa na mkojo bila ridhaa pale haja ndogo inapobana sana kwa ghafla, hali inayoashiria uhitaji kukimbilia msalani kwa ajili ya kujisaidia? |  |  |  |  |
| 4. Haja ndogo inatoka unapokohoa, kupiga chafya au unapofanya zoezi/kazi yeyote? |  |  |  |  |
| 5. Unapata shida kutoa mkojo wote wakati wa haja ndogo? (Unahisi kubakiza mkojo baada ya kukojoa?) |  |  |  |  |
| 6.Unapata maumivu yoyote wakati wa haja ndogo? |  |  |  |  |
